# How Is WHO Responding to Global Public Health Threats?

**DOI:** 10.1371/journal.pmed.0040197

**Published:** 2007-05-29

**Authors:** The PLoS Medicine Editors

## Abstract

The editors discuss two recent WHO initiatives on preparing for and responding to global public health threats: the WHO Rapid Advice Guidelines Group and the new revision of the International Health Regulations.

It is a favored pastime of medical editors and journalists (including ourselves) to criticize the World Health Organization (WHO) for, among other things, its “fossilized bureaucracy,” its lack of clear direction and priorities, the dysfunctional relationship between its headquarters and regional offices, and its faltering steps towards building partnerships [1,2]. But with the arrival of so many new players on the global health scene, and the subsequent fragmentation of global health governance, we surely need WHO more than ever [3]. It is the best placed of all health agencies to coordinate the disparate, often overlapping global health initiatives and to set global norms and standards in health care, and its convening power (its ability to bring together experts) is unparalleled. Two recent WHO initiatives on preparing for and responding to global public health threats show the organization at its best, although there are, as always, some important caveats.

The first initiative, described by Holger Schünemann and colleagues in this issue of *PLoS Medicine*, is the development and pilot testing of the new WHO Rapid Advice Guidelines Group [4]. WHO's standard process for guideline development typically takes years of consultations and endless rounds of revisions, a process that is wholly inadequate for dealing with emergency health threats such as severe acute respiratory syndrome (SARS) or H5N1 avian influenza. In response to requests for advice from frontline clinicians and public health professionals on how to treat H5N1 infections, WHO convened a new type of panel to issue rapid, evidence-based advice. It took one month to convene an expert team, and five weeks for the team to summarize the evidence and prepare draft guidelines. The guidelines were then discussed at a two-day meeting, after which a draft manuscript for publication was prepared within 10 days.

In WHO terms, this time scale was miraculously quick, but there is clearly room for improvement—an even quicker time frame is likely to be needed for future health emergencies. The process could be expedited, say the authors, by identifying or establishing collaborating centers skilled in producing evidence-based guidelines and by building in-house capacity to reduce the time needed to organize a review team. It will also be vital for this new rapid advice group to make its process of panel selection as transparent as possible if the group is to garner the trust of the public health community. WHO has previously come under fire for allowing industry to unduly influence its expert committees, and for failing to ensure that committee members declare their competing interests [3]. The rapid advice group must not sacrifice transparency in the interests of urgency.

We surely need WHO more than ever.

The second initiative is the long overdue 2005 revision of the International Health Regulations (IHR 2005), which comes into force next month [5]. These regulations are an international legal instrument designed to ensure maximum protection against the international spread of infectious disease while minimizing restrictions on travel and trade. Up until the 2005 revision, the regulations in force since 1969 (IHR 1969) required notification of just three diseases: yellow fever, cholera, and plague. The instrument was therefore hopelessly outdated for dealing with the new infectious diseases that have emerged at a rate of about one per year over the last 20 years [6], or indeed for dealing with established threats such as pandemic influenza. IHR 1969 had several other limitations—for example, surveillance relied totally upon individual governments notifying WHO and there were no specific strategies to help build the capacity of resource-poor countries to monitor or respond to outbreaks [7]. Compliance was poor, in part because countries feared that reporting of outbreaks would lead to unwarranted and damaging travel and trade restrictions [5]. China's initial reluctance to disclose the SARS pandemic was motivated by such fears [7].

Under IHR 2005, WHO member states are now required to notify WHO of “all events which may constitute a public health emergency of international concern,” which can include noninfectious events (such as chemical or radiation hazards) [5]. A new algorithm has been devised to aid states in determining what constitutes such an emergency. Criteria for reporting are whether the public health impact of the event is serious, whether it is unusual or unexpected, whether there is a risk of international spread, and whether there is a risk of international restrictions to trade and travel. The algorithm includes a long list of specific diseases that must always be notified, such as viral hemorrhagic fevers, SARS, and human influenza caused by a new subtype.

IHR 2005 requires all states to develop “core surveillance and response capacities” and requires WHO to assist in this development process. Each country must now have a “National IHR Focal Point” to maintain communications between WHO and the member state. When an event is reported, WHO will guide the appropriate response by issuing time-limited recommendations to the member state tailored to the assessed risk of the event. An important new feature of IHR 2005 is that WHO can now use information about health emergencies not just from governments but from a range of sources, including nongovernmental organizations and the media. WHO can also raise the alarm itself about an emergency even when a country has not voluntarily notified the organization.

IHR 2005 is undoubtedly “a great step forward for international public health practice” [8]. On paper at least, the radical revision to the IHR, which took ten years to finalize, gives WHO the teeth it needs to prepare for and respond to any global health threat. But WHO will have to address several important concerns if IHR 2005 is to become a real force for strengthening our collective defenses against public health threats.

The first, and most obvious, concern is that many developing countries lack the financial resources to build core surveillance and response capacity. These countries will be unable to comply with IHR 2005 through no fault of their own. Unless WHO helps to mobilize new funding, an upgraded global surveillance and response system will remain just an aspiration. Donors have taken an interest in preparing for at least one “public health emergency of international concern”—pandemic influenza—but without a way of ensuring equitable distribution of funds, it is the donor countries themselves that will largely benefit from these extra resources. For example, in December 2005 the United States congress allocated $US3.8 billion to help prepare for the next pandemic, of which $US3.3 billion went to the Department of Health and Human Services [9]. Three-quarters of this departmental funding is allocated to the stockpiling of antiviral drugs and vaccines for use in the US, while only 3.8% is dedicated to “international activities.” Poor countries are understandably concerned that the stockpiling of tools for pandemic influenza control will be the preserve of the rich world.

A related concern is that IHR 2005 appears to have no remit to help developing countries deal with *national* public health emergencies. The event must be “of international concern” for the IHR machinery to kick in. It would arguably be better for a country to adopt a precautionary principle rather than to wait until a disease has crossed its borders to become an “international” epidemic. The woolly language of IHR 2005 also leaves the regulations open to the criticism that they are there simply to prevent infectious diseases of the poor world from encroaching upon rich countries.

There are other potential barriers to the success of IHR 2005. The division of power within federations may make it difficult for them to meet the surveillance and reporting requirements of IHR 2005 [10]. It is unclear whether the instrument will have any power to assist states that are not members of WHO, such as Taiwan, which suffered a major SARS outbreak in 2003 and which is at risk of pandemic influenza. It is too soon to tell how IHR 2005 will interact with other guidelines on public health emergencies. The European Union, for example, already has its own network for reporting unusual events that may constitute a public health emergency [11]—does this network supersede the IHR 2005? And it is too soon to tell whether IHR 2005 gives countries enough of an incentive to report epidemics or whether compliance will be just as poor as with IHR 1969.

IHR 2005 has been hailed as “a governance regime unlike anything in the history of international law on public health” [7]. Margaret Chan, WHO's Director-General, believes that the new regulations give the organization the preemptive powers it needs to detect an outbreak early and stop it at its source [12]. IHR 2005 certainly gives the health community a new tool that could promote collective action against global health threats, but the tool will be weakened unless the technical, logistical, and, most crucially, financial hurdles are overcome.

## Abbreviations

IHR: International Health Regulations
SARS: severe acute respiratory syndrome
WHO: World Health Organization
